# Interfacing of Science, Medicine and Law: The Stem Cell Patent Controversy in the United States and the European Union

**DOI:** 10.3389/fcell.2015.00071

**Published:** 2015-11-10

**Authors:** Sonya Davey, Neil Davey, Qian Gu, Na Xu, Rajet Vatsa, Samir Devalaraja, Paul Harris, Sreenivas Gannavaram, Raj Dave, Ananda Chakrabarty

**Affiliations:** ^1^Department of Geography, University of CambridgeCambridge, UK; ^2^Perelman School of Medicine at the University of PennsylvaniaPhiladelphia, PA, USA; ^3^Harvard UniversityCambridge, MA, USA; ^4^The George Washington University School of LawWashington, DC, USA; ^5^Pillsbury Winthrop Shaw Pittman, LLPWashington, DC, USA; ^6^Division of Emerging and Transfusion Transmitted Diseases, Center for Biologics Evaluation and Research, US Food and Drug AdministrationBethesda, MD, USA; ^7^Microbiology and Immunology, College of Medicine, University of Illinois at ChicagoChicago, IL, USA

**Keywords:** stem cell patent elibility, stem cell patent ethics, human embryo, stem cell commericalization, legal interpretation

## Abstract

The patent eligibility of stem cells–particularly those derived from human embryos–has long been under debate in both the scientific and legal communities. On the basis of moral grounds, the European Patent Office (EPO) has refrained from granting patents for stem cells obtained through the destruction of human embryos. On the contrary, the United States Patent and Trademark Office (USPTO) has historically granted patents regarding the isolation and use of human embryonic and other stem cells. To date, these US patents remain valid despite an increasing onslaught of challenges in court. However, recent precedents established in US courts significantly narrow the scope of patent eligibility within biotechnology. This article compares the implications of recent legal changes on stem cell patent eligibility between the EU and US.

## Introduction

Human stem cells have been a focus of research because of their ability to self-renew. Indeed, since the discovery of stem cells over half a century ago, more than 5000 US clinical trials have utilized stem cells (ClinicalTrials). Human stem cells fall into three primary categories: human embryonic stem cells (hESCs), induced pluripotent stem cells (iPSCs), and human parthenogenetic stem cells (hpSCs). hESCs are derived from human blastocysts. iPSCs do not rely on destruction of human embryos and are instead generated directly from adult somatic cells (Takahashi and Yamanaka, 2006) by “reprogramming” them into a pluripotent state. hpSCs are relatively new and formed by *parthenogenesis* (e.g., by chemically stimulating unfertilized oocytes), which also does not involve destruction of human embryos. The oocytes are not fertilized and no viable embryo is created or destroyed. Each of these types of stem cells has unique legal and ethical considerations regarding patent eligibility. Further, the laws in the US and EU on patent eligibility of stem cells are not fully settled and have significant differences. This paper will first summarize the recent changes in EU and US biotechnology patent law, and subsequently compare the implications of the changes in these jurisdictions.

## The EU law on patent eligibility of stem cells

Patent eligibility of human stem cells faces resistance in the EU on morality grounds.

Directive 98/44/EC on the Legal Protection of Biotechnological Inventions (the “Biotech Directive”) regulates the legal protection of biotechnological inventions across the EU. The Biotech Directive prohibits patenting uses of human embryos for industrial or commercial purposes on a morality ground (Directive 98/44/EC, Article 6(2)(c)). The *EPO's Enlarged Board of Appeal* (EBoA) applied the Biotech Directive and ruled that claims directed to products which, at the filing date, could be prepared *exclusively* by a method *necessarily* involving the destruction of human embryos are not patent eligible, even if the said method is not part of the claims [G2/2006 WARF, 2009 OJ EPO 306 (the “WARF decision”)]. The impact of the EBoA decision thus depends on the definition of “human embryo” under the Biotech Directive.

In *Brüstle v. Greenpeace* (*Brüstle v. Greenpeace eV*, 2012), the Court of Justice of the European Union (CJEU) interpreted the term “human embryo.” The CJEU included into the scope of “human embryo” not only fertilized human ovum, but also:

“*…non-fertilised human ovum whose division and further development had been stimulated by parthenogenesis. Although those organisms have not, strictly speaking, been the object of fertilisation, due to the effect of the technique used to obtain them they are, as is apparent from the written observations presented to the Court, capable of commencing the process of development of a human being just as an embryo created by fertilisation of an ovum can do so*.”

(Emphasis added). As a result of this interpretation, the CJEU held that a claim directed to “A cell culture comprising primate embryonic stem cells” was patent ineligible under the Biotech Directive. Directly following this decision, scientists were anxious that funding for stem cell research and market competitiveness for stem cell therapies would decrease in EU (Abbott, 2011).

The CJEU apparently drew the line around “human embryo” by the capability of “commencing the process of development of a human being.” However, the capability is not fixed but may change over time when new scientific discoveries make tissues previously not capable of “commencing the process of development of a human being” capable of doing so.

This changing scope of “human embryo” due to new scientific development was recently manifested in a case before the CJEU. International Stem Cell Corporation (ISCC) applied in the UK for two patents relating to methods where parthenogenesis is used to activate a human oocyte. Using the *Brüstle* decision as a precursor, the UK Patent Office concluded that because the parthenogenetically derived structure (parthenote) was analogous to the blastocyst stage of normal embryonic development, this fell within the definition of “human embryo,” and was thus excluded from patentability. A parthenote is an unfertilized egg chemically induced through a process called parthenogenesis to begin developing as if it had been fertilized, and behaves like an embryo in early development.

ISCC appealed to the English High Court questioning the clause—“capable of commencing the development of a human being (*International Stem Cell Corporation v. Comptroller General of Patents, UK*, 2013).” ISCC argued that a parthenogenetically stimulated human oocyte was not capable of producing an embryo due to its inherent biological limitations, explaining that a parthenote contains only the maternal nuclear chromosome but no paternal DNA and is known to not undergo full development to give rise to an embryo.

It is clear from the CJEU's analysis of the *Brüstle* case that at the time, scientific knowledge stated that an unfertilized human ovum whose division and further development had been stimulated by parthenogenesis *did* have the capacity to develop into a human being. However, current scientific knowledge has established that mammalian parthenotes cannot develop into viable human beings because they lack the paternal DNA necessary for the development of extra-embryonic tissue (Brevini et al., 2008). Human parthenotes have been shown to develop only to the blastocyst stage over about five days. Thus, on December 18, 2014, the CJEU concluded “that unfertilized human ovum whose division and further development had been stimulated by parthenogenesis does not constitute a ‘human embryo’” (*International Stem Cell Corporation v. Comptroller General of Patents, CJEU*, 2014). By narrowing the definition of “human embryo,” the CJEU indirectly reduced the reach of the WARF decision of the EBoA and the *Brüstle* decision and opened the door for patenting hpSCs.

The ISCC decision differentiating a parthenote from an embryo invites at least two questions. First, if a human parthenote is not a potential human life but a human embryo is, what exactly is the characteristic of life that is present in the embryo formed from blastocyst during fertilization but not in the parthenote-derived blastocyst from which stem cells are harvested, disregarding paternal DNA contribution? Second, what is the definition of “human”? Since genetically-engineered humans cannot be patented either in the EU or in the US, but genetically-manipulated animals (e.g., oncomouse) can be patented in the US, what fundamentally is the difference between an animal, such as a monkey, and a human being? This is not just a rhetorical question, since a patent application for a part-human part animal-chimera was filed to the USPTO in 1998 but rejected on the grounds of the 13th amendment of the US Constitution prohibiting slavery and ownership of human beings (Chakrabarty, 2003). Nevertheless, the pertinent question, both moral and legal, is how many human characteristics, including certain number of human genes, must be present in an animal to give it the legal status of a human? The problem is more acute in the EU where the *ordre public* and morality clause prevent patenting of not only humans but also human cells or organs.

In summary, though the ISCC decision allows patenting of both iPSCs and parthenotes, (as these cells are incapable of becoming human beings), the EU still remains strict on its policy against patent protection of hESCs.

## The US law on patent eligibility of stem cells

In contrast to that of the EU, US law poses no morality-based barrier to patenting human stem cells Public Law (1996). In the US, patent eligible subject matter is “any new and useful process, machine, manufacture, or composition of matter, or any new and useful improvement thereof.” 35 USC §101. Although the US Supreme Court noted that “Congress plainly contemplated that the patent laws would be given wide scope,” the Court has created three exceptions to patent eligible subject matter: laws of nature, natural phenomena, and abstract ideas (*Diamond v. Diehr*, 1981; USPTO, 2014).

For a particular kind of human stem cell to be eligible for patents, it must not fall under any of the three exceptions, the most relevant of which is the natural phenomena exception. In *Diamond v. Chakrabarty*, the US Supreme Court clarified that merely being alive is not sufficient to exclude an invention from patent eligible subject matter under §101 (*Diamond v. Chakrabarty*, 1980). Namely, merely being alive does not cast an invention into the natural phenomena exception. Stem cells have been patented in the US under this pretense for the past thirty years.

On the other hand, with regards to the government funding of human embryonic stem cell research, from 1996 to 2009, the Dickey-Wicker Amendment of 1996 prohibited the use of government funds for any research that destroyed and created human embryos (Zachariades, 2013). In 2009, President Obama's Executive Order allowed the funding of research that employed embryos, if these embryos were created during *in vitro* fertilization for reproductive purposes but were no longer needed for such purposes (Zachariades, 2013).

The recently enacted Leahy-Smith America Invents Act (AIA) and influential Court decisions regarding BCRA1/2 and cell-free fetal DNA may significantly limit, however, the scope of biotechnology patent eligibility in the US. These dramatic changes in the law may directly oppose the vision of Shinya Yamanaka, a strong believer in the importance of stem cell patenting to promote research, drug discovery, regenerative medicine, and global access (Yamanaka, 2015).

### AIA

The AIA, passed in September 2011, provides that “no patent may issue on a claim directed to or encompassing a human organism (Leahy-Smith America Invents Act (AIA), Pub. L. 112-29, sec. 33(a), 125 Stat. 284).” The AIA is the most significant patent change in the US since 1952. The legislative history of AIA clarifies that under this act, stem cells are patent eligible but patent claims directed to or encompassing a human organism, including human embryos are prohibited (157 Cong. Rec. E1177-04 Testimony of Representative Dave Weldon previously presented in connection with the Consolidated Appropriations Act, 2004, Pub. L. 108-199, ′634, 118 Stat. 3, 101, and later resubmitted with regard to the AIA; see 149 Cong. Rec. E2417-01). The AIA, however, does not define the term “human organism.” In the future, if the US Supreme Court interprets “human stem cells” as being a kind of “human organism,” stem cells would be patent ineligible. After this ruling, in late 2011, Geron–a biotechnology company that develops and commercializes therapeutics for cancer by inhibiting telomerase—announced an abandonment of hESC research (Torrance, 2013).

### Myriad

In *Association for Molecular Pathology v. Myriad Genetics, Inc*. (2013), hereafter referred to as the *Myriad* decision, the Supreme Court ruled that cDNAs are patent eligible, but isolated DNAs are not. The case revolved around two important societal issues: breast cancer and gene patenting. In the mid-90s, Myriad Genetics Inc. successfully sequenced and developed a comprehensive diagnostic test for hereditary breast cancer based on mutations in the BRCA1 and BRCA2 genes. Myriad's US Pat. No. 5,747,282 claimed, “An isolated DNA coding for a BRCA1 polypeptide, said polypeptide having the amino acid sequence set forth in SEQ ID NO:2.”

As per the *Myriad* decision, DNA means genomic DNA that has been “isolated” from the body, and “isolated” means merely removed and separated “from the surrounding genetic material”; cDNA means “synthetically created DNA…which contains the same protein-coding information found in a segment of natural DNA [exons] but omits portions within the DNA segment that do not code for proteins [introns].” The *Myriad* decision explains “what is *not* implicated by this decision” and states, “We merely hold that genes and the information they encode are not patent eligible under §101 simply because they have been isolated from the surrounding genetic material.” But notwithstanding the disclaimer, the lower courts may apply the *Myriad* decision to imply that if a stem cell is closer to being merely “isolated” from the body, the stem cell is likely not patent eligible. Conversely, if a stem cell is a synthetically created stem cell that contains the same information found in a natural stem cell but omits portions within the natural stem cell, e.g., portions that cannot replicate, then the synthetically created stem cell is likely patent eligible.

The *Myriad* decision appears to undermine the patent eligibility of hESCs because isolated and purified hESCs are essentially identical to hESCs in human blastocysts. Such a contention resonates with many people who are either against patenting any part of the human body or interfering in the natural processes of human development.

In fact, Consumer Watchdog used AIA and *Myriad* as the basis to challenge WARF and thus appeal to the courts to repeal patents on isolated stem cells. However, in June 2014 the Federal Circuit Court said that it was required that there be an “actual case or controversy”—because WARF never sued Consumer Watch, the courts said the group did not have standing to appeal (Sherkow and Scott, 2015). This demonstrates that in the future, other patent petitioners on behalf of public health (like in the *Myriad* decision) will likely attempt to repeal patent protection of isolated stem cells.

It does not matter, for the purposes of defining something as a product of nature, whether the product contains only one or millions of nucleotides or amino acids. What matters is whether the nucleotides or amino acids are in their natural form that exists in the body, other than having been isolated from their natural environment. If so, then these products are not patent eligible. If, however, the products are not only isolated from their natural environment, but also purified such that they are no longer in their natural form (such as by removal of introns), then such purified and isolated products are patent eligible.

### Sequenom

A more recent decision in June 2015 further supported this trend of legal change with respect to patent eligible subject matter in biotechnology. In *Ariosa Diagnostics Inc. v. Sequenom Inc*. (2015) (hereafter referred to as the *Sequenom* case), the US Court of Appeals for the Federal Circuit invalidated Sequenom Inc.'s patents on methods of using cell-free fetal DNA (cffDNA)—a naturally occurring extracellular fetal DNA that circulates in the blood stream of an expecting mother–for the efficient detection of fetal genetic defects. Although the court acknowledged that the Sequenom claims represented an important scientific breakthrough, the fact that cffDNA in the maternal blood is a natural phenomenon is indisputable. Moreover, the Federal Circuit concluded that the routine and well understood methods of DNA detection were not sufficient to transform the claimed subject matter into a patentable application. In the US, a method claim is patentable if either the composition of matter in the claims is patent eligible, novel, and non-obvious or the method steps are novel and non-obvious. In *Sequenom*, the courts decided that cffDNA (composition) was not patent eligible and that the methods of detection were obvious.

These cases bring to question—what extent of man-made alteration is necessary for a stem cell to not simply be considered an isolated form? *Myriad* demonstrates that while cDNA is patentable, isolated DNA is not; it is unclear how these cases will affect the patentability of stem cells in US.

## Implications

In the EU, it appears likely that the morality clauses excluding the patent eligibility of hESCs will remain in effect. The EU's narrowed patentability for hESCs was thought to be a potential obstacle to hESC commercialization in Europe in comparison to the US (Porter et al., 2006).

In the US, however, the fate of hESC patents is tremendously uncertain. As explained in the above sections, as the US significantly narrows the scope of patent eligible subject matter in biotechnology, stem cells face the risk of becoming classified as patent ineligible subject matter. Despite the absence of a morality clause, one could foresee the US hESC patent policies approaching those of the EU.

Indeed, the dramatic change in the law in recent years may set the stage for how future issues will be decided in the US. The next center of debate may well be the patents regarding hESCs held by WARF. WARF US Patent No. 7,029,913 relates to a replicating *in vitro* cell culture of human embryonic stem cells. The WARF patents have been challenged on grounds of patent eligibility by Consumer Watchdog and the Public Patent Foundation. At the core, the prosecutors categorize the hESC patents as naturally occurring phenomenon. In this light, comparisons are made between the original stem cells and naturally occurring DNA, and the cultured stem cells and artificial cDNA. Thus far, the patents have survived at the US Court of Appeals of the Federal Circuit, which rather than ruling on the validity of the patents, ruled that as a third party not directly harmed by the decision, Consumer Watchdog did not have the legal standing, but this could change (*Consumer Watchdog v. Wisconsin Alumni Research Foundation*, 2014).

Looking ahead, the curtain is far from being closed. If the right group with standing litigates and succeeds in spurring a widespread social movement, it is unknown whether hESC patents will survive in the US. Indeed, the convergence of US and EU laws regarding stem cell patent eligibility may be inevitable.

### Conflict of interest statement

The authors declare that the research was conducted in the absence of any commercial or financial relationships that could be construed as a potential conflict of interest.
